# Determination of the Clean Air Delivery Rate (CADR) of Photocatalytic Oxidation (PCO) Purifiers for Indoor Air Pollutants Using a Closed-Loop Reactor. Part II: Experimental Results

**DOI:** 10.3390/molecules22030408

**Published:** 2017-03-06

**Authors:** Valérie Héquet, Frédéric Batault, Cécile Raillard, Frédéric Thévenet, Laurence Le Coq, Éric Dumont

**Affiliations:** 1 UMR CNRS 6144 GEPEA, IMT Atlantique, La Chantrerie, 4 rue Alfred Kastler, CS 20722, 44307 Nantes CEDEX 3, France; valerie.hequet@imt-atlantique.fr (V.H.); frederic.batault@mines-nantes.fr (F.B.); cecile.raillard@univ-nantes.fr (C.R.); laurence.le-coq@imt-atlantique.fr (L.L.C.); 2 IMT Lille-Douai, Université de Lille, SAGE, F-59000 Lille, France; frederic.thevenet@imt-lille-douai.fr

**Keywords:** photocatalysis, Clean Air Delivery Rate (CADR), indoor air quality, Volatile Organic Compounds (VOCs), air cleaner

## Abstract

The performances of a laboratory PhotoCatalytic Oxidation (PCO) device were determined using a recirculation closed-loop pilot reactor. The closed-loop system was modeled by associating equations related to two ideal reactors: a perfectly mixed reservoir with a volume of V_R_ = 0.42 m^3^ and a plug flow system corresponding to the PCO device with a volume of V_P_ = 5.6 × 10^−3^ m^3^. The PCO device was composed of a pleated photocatalytic filter (1100 cm^2^) and two 18-W UVA fluorescent tubes. The Clean Air Delivery Rate (CADR) of the apparatus was measured under different operating conditions. The influence of three operating parameters was investigated: (i) light irradiance I from 0.10 to 2.0 mW·cm^−2^; (ii) air velocity v from 0.2 to 1.9 m·s^−1^; and (iii) initial toluene concentration C_0_ (200, 600, 1000 and 4700 ppbv). The results showed that the conditions needed to apply a first-order decay model to the experimental data (described in Part I) were fulfilled. The CADR values, ranging from 0.35 to 3.95 m^3^·h^−1^, were mainly dependent on the light irradiance intensity. A square root influence of the light irradiance was observed. Although the CADR of the PCO device inserted in the closed-loop reactor did not theoretically depend on the flow rate (see Part I), the experimental results did not enable the confirmation of this prediction. The initial concentration was also a parameter influencing the CADR, as well as the toluene degradation rate. The maximum degradation rate r_max_ ranged from 342 to 4894 ppbv/h. Finally, this study evidenced that a recirculation closed-loop pilot could be used to develop a reliable standard test method to assess the effectiveness of PCO devices.

## 1. Introduction

Indoor Air Quality (IAQ) is a current problem because so many people spend time indoors and are exposed to numerous pollutants, such as Volatile Organic Compounds (VOCs). To overcome this issue, PhotoCatalytic Oxidation (PCO) appears as an efficient technology for air cleaning [1,2]. However, the performances of PCO cleaners in development at the laboratory scale or already available on the market have to be evaluated for critical comparison [3]. Consequently, there is a need to develop reliable methodologies to assess the effectiveness of such cleaners and standard test methods prior to their coming onto the market [4,5]. In Part I of the paper, the performance of any PCO device inserted in a closed-loop recirculation system was investigated through a theoretical model. This model allows the Clean Air Delivery Rate (CADR), considered a useful tool to evaluate the air cleaning capacity of a PCO device, to be determined experimentally [6]. The higher the CADR value, the faster the PCO device removes the primary pollutants. As a result, a recirculation closed-loop system can be used (i) to assess the influence of operating parameters and (ii) potentially to compare PCO units. Therefore, the aim of this part of the paper is to check experimentally that the model can be used effectively to determine the CADR of a PCO laboratory device under different operating conditions (pollutant concentration, light irradiance intensity and flow rate). A more accurate analysis of the results using the proposed model leads to a discussion of the main influential parameters. Toluene was chosen as a representative VOC target because it is usually encountered in indoor air at concentrations of around a few tens of parts per billion by volume (ppbv). It is also a common VOC used to study PCO devices found in the literature and in different standard tests.

## 2. Model

**Figure 1:**
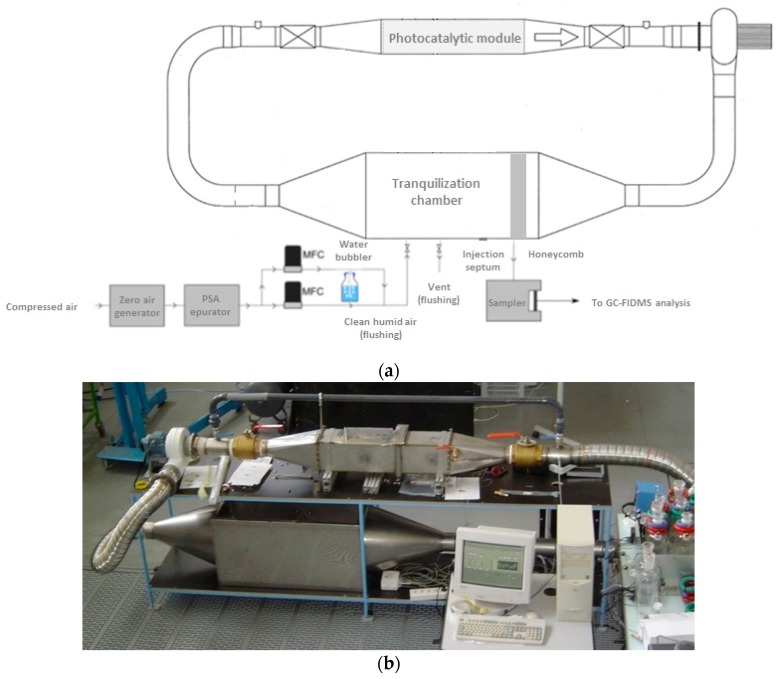
Experimental closed-loop reactor operating in recirculation mode. (**a**) Schematic representation; and (**b**) photograph of the whole system.

The mathematical model, extensively described in Part I, is based on the one proposed by Walker and Wragg [7] for concentration-time relationships established for recirculating electrochemical reactor systems. The closed-loop reactor operating in recirculation mode (Figure 1) was modeled by associating two ideal reactors: a perfectly mixed reservoir with a volume V_R_ and a plug flow system corresponding to the PCO device with a volume V_P_. Considering that the volume of the PCO device is very small in relation to the volume of the reservoir (i.e., if the ratio of the residence times (τ_P_/τ_R_) tends to zero), the concentration-time relationship is:

(1) $$C=C_{0}\;\exp\left\{-\frac{t}{\tau_{R}}\left[1-\exp\left(-\alpha \right)\right]\right\}$$

Equation (1) should be able to describe satisfactorily the decrease in the pollutant concentration over time provided that the total time of the experiment does not exceed 2 h. In this equation, the term α (= k τ_p_) is the fractional yield of the treated flow rate of the PCO device. In other words, α corresponds to the percentage of the total flow rate treated during the time τ_R_ (i.e., during one cycle). As developed in Part I, the term α can be related to the parameter CADR (Clean Air Delivery Rate) typically used to evaluate the air cleaning capacity of a PCO reactor. The CADR is defined as the product of the device efficiency and the volumetric air flow rate through the apparatus [8,9]:

(2) CADR=α Q

Since the volume of the PCO device is small in relation to the volume of the reservoir, it is expected that the CADR value is small with regard to the air flow rate Q, and consequently, α does not exceed some %. In this case, Equation (1) can be simplified as:

(3) $$C=C_{0}\;\exp\;\left(-t\frac{\alpha }{\tau_{R}}\right)\;=C_{0}\;\exp\;\left(-t\frac{\mathrm{CADR}}{V_{R}}\right)$$

Thus, the decrease in pollutant concentration follows a first-order decay model. The value of the parameter α is then directly deduced from the slope of the curve ln(C/C_0_) vs. t. Rearranging Equation (3), the overall efficiency of the PCO device to treat the air is given by Equation (4), where τ_R_/α is the time constant (t_c_) of the closed-loop reactor (i.e., the time needed to reach a 63.2% conversion of pollutant) and 1/α is the cycle number needed to obtain E = 0.632.

(4) $$E=\frac{C_{0}-C}{C_{0}}=1-\exp\;\left(-t\frac{\alpha }{\tau_{R}}\right)=1-\;\exp\;\left(-\frac{t}{t_{c}}\right)$$

The maximum degradation rate determined at the beginning of the experiment (at t = 0) is then:

(5) $$r_{\max}=C_{0}\frac{\alpha }{\tau_{R}}\;=C_{0}\frac{\mathrm{CADR}}{V_{R}}$$

## 3. Experimental Methods

### 3.1. Recirculation Closed-Loop System

The closed-loop (batch) system used in this study is described in Figure 1. As experiments were mainly carried out at concentrations lower than 1 ppmv, the whole pilot (V_R_ = 0.42 m^3^) was constituted of stainless-steel to limit the loss of toluene by adsorption on the walls. The PCO device was installed in an internal duct and operated in recirculation mode. The air flow rate was controlled by a variable speed fan operating inside the closed-loop system. Flow rates were determined from pressure drop measurements between the two sides of a calibrated diaphragm. A tranquilization chamber was used for toluene injection and gas sampling. A honeycomb part located at the inlet of the chamber provided a homogeneous flow distribution in the reservoir. In such conditions, the polluted air could be considered perfectly mixed in the reservoir.

### 3.2. PCO Device

**Figure 2:**
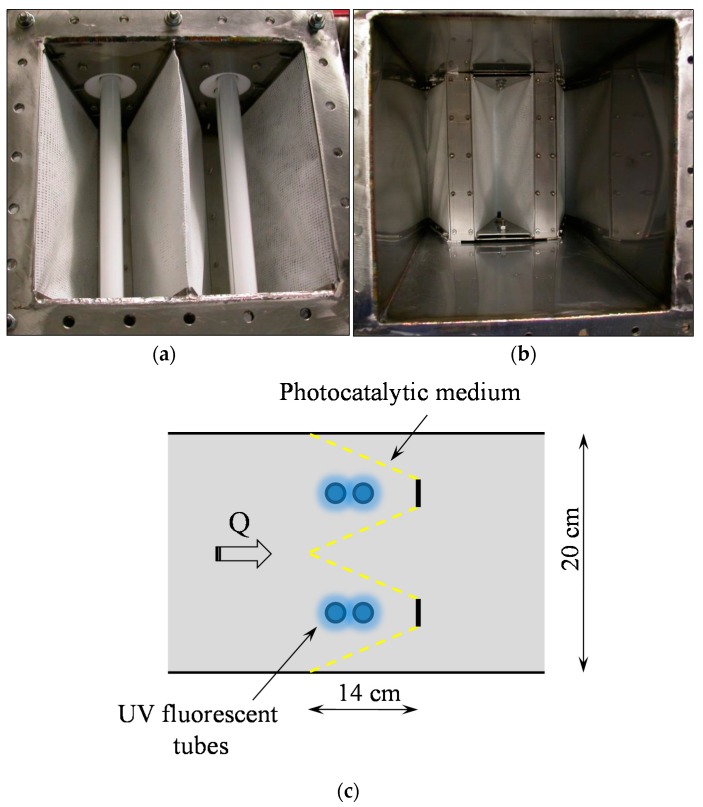
PhotoCatalytic Oxidation (PCO) device. View of: (**a**) the inlet side; (**b**) the outlet side; and (**c**) diagram of the pleated filter configuration.

The PCO device was composed of a photocatalytic medium and UV lamps (Figure 2). The photocatalytic medium (Ahlström Paper Group) consisted of a thin fibrous support (250 µm thick) composed of a mixture of cellulose, polyester and polyamide and coated with P25 TiO_2_ in a SiO_2_ binder. The TiO_2_ load was 17 g·m^−2^. This medium was specifically produced for these experiments. The BET specific surface area of the medium (31 ± 1 m^2^·g^−1^) was measured by a Micromeritics ASAP 2010 device (Micromeritics Instrument Corp., Norcross, GA, USA). A pleated geometry filter was used in order to increase the surface implemented in the reactor for air treatment. Due to this geometry, the total surface area of the photocatalytic medium was 1100 cm^2^, and the corresponding mass of TiO_2_ was 1.87 g. Two 18-W UVA UV fluorescent tubes (Philips PL-L series, Koninklijke Philips N.V., Amsterdam, The Netherlands) were placed inside the filter folds as illustrated in Figure 2. Taking into account the pleated configuration of the PCO device, its overall volume V_P_ was 5.6 L (0.2 × 0.2 × 0.14 = 5.6 10^−3^ m^3^), corresponding to 1.3% of the whole pilot. In order to control the irradiance received by the photocatalytic medium, a variable voltage supply was used to change the light intensity provided by the lamps. Before experiments, light irradiance was calibrated using a Vilber Laurmat VLX 3-W radiometer equipped with a calibrated CX-365 sensor (Vilber Lourmat Deutschland GmbH, Eberhardzell, Germany). The calibration procedure is extensively described in Batault et al. [10].

### 3.3. VOC Generation and Analytical Methods

To prepare the polluted gas phase at the desired concentrations, liquid toluene (99.99% purity, purchased from VWR International, Fontenay sous Bois, France) was evaporated in a 1-L glass reactor at room temperature. The toluene gaseous phase was then injected into the tranquilization chamber of the pilot by means of a 50-mL syringe through a septum (Figure 1). The decrease in toluene concentration over time in the photocatalytic reactor was monitored during the experiments using a Markes FLEC Air Pump 1001 sampler (Markes International Ltd., Llantrisant, UK) at room temperature and at a flow of 100 mL/min. It consisted of an off-line sampling on multi-sorbent cartridges containing three beds of Supelco activated carbon: Carbopack B, Carbopack C and Carbopack X. Sorbent tubes were then analyzed using a TD/GC/FID/MS system (Thermal Desorption/Gas Chromatography/Flame Ionization Detector/Mass Spectrometer, Perkin-Elmer, Lyon, France). The gas chromatograph was equipped with a 60-m Rxi-624Sil MS non-polar column from Restek, which was simultaneously connected to two detectors: (i) a Flame Ionization Detector (FID) for the quantification of compounds; and (ii) a Mass Spectrometer (MS) for chromatographic peak identification. A calibration curve for toluene quantification was carried out by diluting a standard toluene cylinder (1040 ppbv in N_2_, Prax’air) with clean air. The detection limit was estimated to be lower than 21 ppbv. Three experiments were performed in identical experimental conditions (I = 0.35 mW·cm^−2^, v = 0.6 m·s^−1^, C_0_ = 600 ppbv) to assess the experimental error in the determination of pollutant concentrations. As evidenced in the paper by Batault et al. [10], the degradation curves of the three experiments indicated that the results were repeatable. The experimental error in the determination of the initial concentration C_0_ was 17%, which corresponds to the value given in Destaillats et al. [1] for a pleated filter (around 15%).

### 3.4. Operating Conditions

Before each experiment, the closed-loop reactor was first flushed with VOC-free air in order to remove traces of VOCs in the system. The photocatalytic material itself is cleaned by irradiation under 50% of relative humidity for several hours. Then, the reactor was flushed again. For experiments, the air entering the reactor was first treated with a zero air generator (Claind 2020, CryoService Limited, Worcester, UK). The CO_2_ and humidity were then removed using a suppressor TDGSi PSA device (F-DGSi, Evry, France). Next, the relative humidity of the air stream in the closed-loop reactor was set using a hygro-transmitter measurement, a room temperature bubbler and according to an appropriate gas dilution by means of two Brooks mass flow controllers. The relative humidity was set at the targeted value, 50%, i.e., 13,000 ppm, to be close to real indoor air conditions (Figure 1).

The influence of three operating parameters (light irradiance I, air velocity v and initial toluene concentration C_0_) was investigated in order to determine the change in the performance of the PCO device. The light irradiance ranged from 0.1 to 2.0 mW·cm^−2^. The air velocity was determined as the ratio between the air flow rate, ranging from 28.8 to 273.6 m^3^·h^−1^, and the cross-sectional area of the PCO device (20 cm × 20 cm square-section duct, i.e., 0.04 m^2^). Thus, the air velocity was varied from 0.2 to 1.9 m·s^−1^. In the present work, the photocatalytic module was inserted in a 20 cm × 20 cm square cross-section so that the hydraulic diameter DH was 20 cm. The Reynolds number thus ranged between 2500 and 26,000, depending on the air velocity value (0.2 to 1.9 s^−1^), and the reaction rate was not limited by mass transfer [11]. In these conditions, the residence time in the reservoir ranged from 52.5 to 5.5 s. Four initial concentrations were selected for experiments: 200, 600, 1000 and 4700 ppbv. The 17 experiments were carried out under the conditions defined in Table 1.

## 4. Results and Discussion

**Figure 3:**
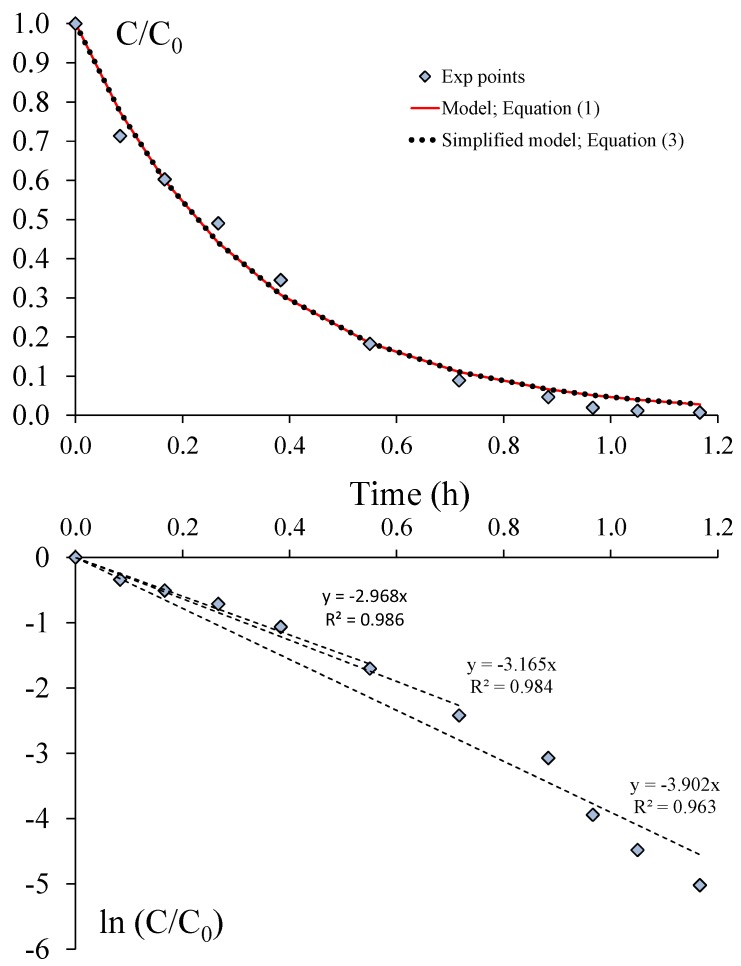
Typical change in toluene concentration over time. Experimental data and models (Exp 10; I = 0.35 mW·cm^−2^; v = 1.0 m·s^−1^; C_0_ = 1000 ppmv).

Figure 3 presents a typical result of changing toluene concentration over irradiation time (corresponding to Exp 10). The experimental C/C_0_ measurements were fitted using both the predictive model (Equation (1)) and the simplified model (Equation (3)). Numerical resolutions were carried out using Excel^®^ solver. The procedure is based on the linear least-squares method, which minimizes the Sum of Squared Residuals (SSR) between experimental and predicted values (for i ranging from one to n, which corresponds to the n concentration values measured during the experiment).

(6) $$SSR=\overset{n}{\underset{i=1}{\sum }}{\left\{{\left(\frac{C}{C_{0}}\right)}_{i}^{exp}-{\left(\frac{C}{C_{0}}\right)}_{i}^{model}\right\}}^{2}$$

According to Figure 3, it can be observed that the predictive model (Equation (1)) described the experimental data satisfactorily within a total time for the toluene degradation experiment of less than two hours. Moreover, as the percentage of the total flow rate treated during one cycle (τ_R_ = 10.5 s) was low (α = 0.9% in the present case; Table 1), the assumption of using the simplified model (Equation (3)) was satisfied, and thus, the first-order decay model could be used for the CADR determination (Figure 3). Nonetheless, for irradiation times longer than 0.7 h, corresponding to a C/C_0_ ratio value lower than 0.15, it appears that the model slightly over-predicts the experimental data (this observation can be made for all experiments as soon as C/C_0_ < 0.20). This result, reported in the literature [12], is clearly highlighted in the curve ln(C/C_0_) vs. time (Figure 3). Such deviations may be due to physical reasons (mass transfer limitation), analytical reasons (accuracy of the measurements when the toluene concentration tends to zero) or mathematical reasons (because lim ln(C/C_0_) tends to −∞ for C/C_0_ tending to zero). Whatever the reasons, which need to be specifically studied, the problem with using the curve ln(C/C_0_) vs. time for the slope determination is choosing the number of points to take into account. As highlighted in Figure 3, the slope is between −2.97 and −3.90 h^−1^ according to the number of points selected, which corresponds to a 35% deviation. These slope values have to be compared with the value obtained by modeling all of the experimental data using Equation (3) and the Excel^®^ solver. For the experiment Exp 10, the term α/τ_R_ corresponding to the absolute value of the slope is 3.08 h^−1^ (α/τ_R_ = 0.0090/(10.5/3600)), which indicates that only the first six or seven points should have been chosen for a correct slope determination. Since the Excel^®^ solver is easily usable for data analysis, such a tool should be preferred for CADR determination using Equation (1) or Equation (3) rather than the analysis of the curve ln(C/C_0_) vs. time. Consequently, all of the results given in the present paper (Table 1) came from the analysis of all of the experimental data using Equation (1).

**Figure 4:**
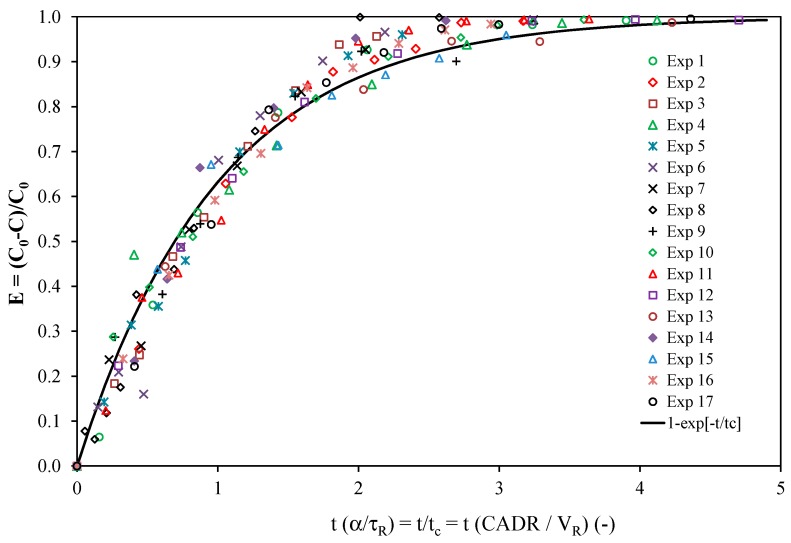
Toluene removal efficiency for all experiments according to the generalized form (Equation (4)).

**Figure 5:**
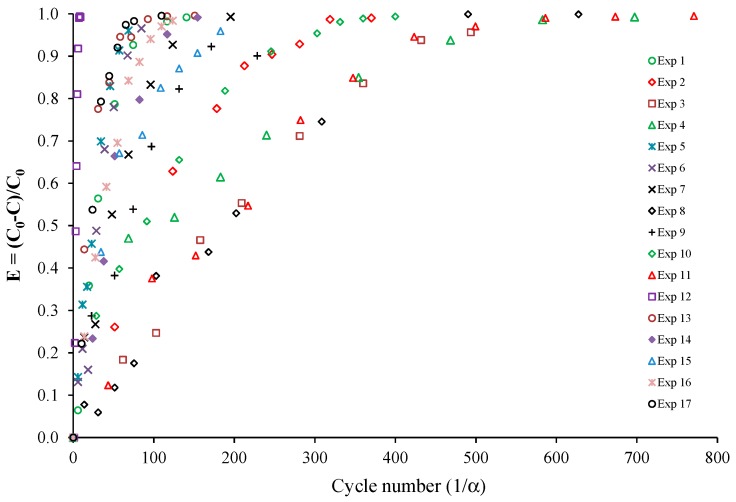
Toluene removal efficiency for all experiments as a function of the number of cycles in the closed-loop reactor.

From Table 1, it can be concluded that the duration of each experiment (corresponding to 5 t_c_) was less than 2 h with the exception of Exp 3 (due to the low value of the light irradiance and high initial concentration) and Exp 8 (because the initial concentration was significantly higher than that of the other experiments, C_0_ = 4700 ppbv). Consequently, experiments can be satisfactorily modeled using Equation (1). For Exps 3 and 8, the total time of the experiment was longer than 3 h, and the approximate solution given by Equation (1) becomes inaccurate as indicated in Part I. From Table 1, it also appears that the maximum α value was 6.4% for Exp 12 and less than 5% for the other experiments. As a result, the simplified model given by Equation (3) can also be used for the analysis of almost all of the experiments, as already highlighted in Figure 3 for Exp 10. Figure 4 and Figure 5 present the toluene removal efficiency (normalized concentrations) for all of the experiments according to the generalized (dimensionless) form and as a function of the number of cycles, respectively (Equation (4)). Figure 4 confirms that irrespective of the operating conditions, the experiments are satisfactorily described by the first-order decay model. Moreover, Figure 5 shows that the number of cycles needed for toluene degradation is strongly dependent on the operating conditions. Such a graphical representation is useful for a comparison at a glance.

### 4.1. CADR Determination

Depending on the operating conditions, the results given in Table 1 show that CADR ranged from 0.35 to 3.95 m^3^·h^−1^, i.e., one order of magnitude for the same PCO device. The time constant t_c_ of the closed-loop reactor varied accordingly, from 392 to 4065 s. Moreover, the number of cycles (1/α) needed to reach a 63.2% conversion of pollutant varied from 16 to 244 corresponding to a ratio of 15. From Table 1, it appears that the performances of the PCO device are strongly dependent on the operating parameters.

Since the experimental data reported in the literature are often obtained under different operating conditions (the design of the reactor or test chamber, flow rate, light intensity, pollutant nature and concentrations), the direct comparison of CADR values given in the literature is therefore not suitable. Nonetheless, the study of CADR values obtained using a similar procedure can be informative. For instance, the curve ln(C/C_0_) vs. t was applied by Costarramone et al. to compare the efficiency of different photocatalytic air purifiers [4]. Studying the toluene degradation performance of eight commercial PCO devices in a 1.2-m^3^ closed-loop chamber, these authors reported CADR values from 0.67 to 24.5 m^3^·h^−1^ for an initial concentration of 1000 ppbv and from 2.35 to 10.94 m^3^·h^−1^ for an initial concentration of 250 ppbv. However, the CADR values were directly calculated assuming a first-order decay, although both assumptions described in Part I of the paper were not necessarily checked. Thus, the authors indicated that the CADR values could be quite inaccurate. Moreover, they reported that the comparison of the PCO devices was not straightforward since so many parameters varied. For instance, the CADR did not follow the order of maximum flow rates of the devices. Consequently, with the aim of developing a standard for the certification of different types of PCO apparatus, there is a need to assess the relative influence of each operating parameter on the performances of the same PCO device.

### 4.2. Effect of the Light Irradiance Intensity

**Figure 6:**
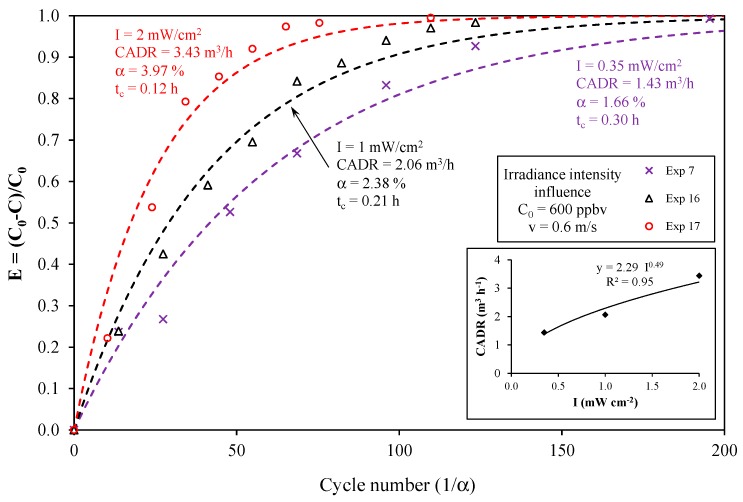
Effect of irradiance intensity on toluene removal efficiency (experimental points and model). Insert: effect of irradiance intensity on the CADR value.

Figure 6 depicts the influence of the light irradiance intensity on toluene removal at a constant flow rate and constant concentration. The greater the light intensity is, the faster the degradation and the higher the CADR are. The degradation rate is varying with light intensity I as I^n^ with n approaching one at very low intensities and n approaching zero at very high values. An intermediate case is often described in the literature where n is around 0.5 [13]. The insert in Figure 6 shows that the relationship between CADR and light intensity is: CADR = 2.3 I^0.49^. Thus, the CADR varies as the square root of the light intensity, which corresponds to the trend usually reported in the literature [14]. In fact, it is generally agreed that the reaction rate increases with increasing light intensity, as the heterogeneous photocatalytic reaction depends on the irradiation of the TiO_2_ surface by UV light to produce electron/hole pairs, even though some of them recombine. Several regimes are defined: a first-order regime in which the electron-hole pairs are consumed more rapidly by chemical reactions than by recombination; and a half-order regime in which the recombination rate dominates [6,15,16]. Nevertheless, it has to be recalled that the CADR is proportional to the overall kinetic rate constant of the apparatus, which can differ from the photocatalytic reaction rate since the reactor dynamics are also taken into account (see Part I). Despite this difference, it can be considered that the CADR determination reflects the actual photocatalytic activity of the PCO device satisfactorily.

### 4.3. Effect of the Flow Rate

**Figure 7:**
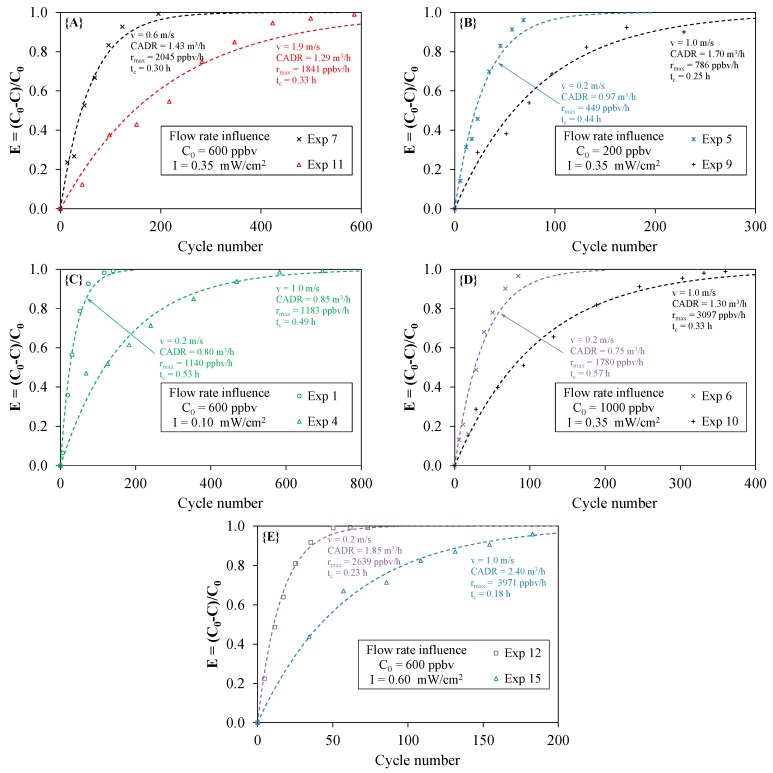
Flow rate influence on toluene removal efficiency according to the initial concentration C_0_ and the light irradiance I (cases **{A}**–**{E}**; experimental points and model; cycle number = 1/α).

As indicated in Part I, the overall efficiency, as well as the maximum degradation rate in the closed-loop reactor do not theoretically depend on the flow rate. In fact, an increase in the flow rate leads to a decrease in the residence time τ_P_ in the PCO device, which reduces the α value, and consequently, many cycles are required for the total conversion of the pollutant. As a result, for a given PCO device, the CADR should be the same irrespective of the flow rate used in the experiment, provided that all other parameters are kept constant. Figure 7 displays five cases illustrating the influence of flow rate for a constant initial concentration and a constant light irradiance. The results are contrasting. For example, cases **{A}** and **{C}** are in agreement with the theory, whereas cases **{B}** and **{D}** indicate that the flow rate had a significant influence on both the CADR and the maximum degradation rate. Moreover, taking into account the accuracy of the experimental measurement, case **{E}** cannot be used to tip the balance. The results from Figure 7 clearly highlight the difficulty of intuitively sensing the effect of the flow rate. It should be noted that a statistical analysis of the results using an experimental design was not sufficient to understand the actual role of the air velocity [10], whereas a previous study carried out at the ppmv level demonstrated a negative influence of the increase in the flow rate on PCO performances [16]. According to Destaillats et al. [1], the contact time of a pollutant on the surface of a PCO medium is one of the most critical parameters for reactor design because a sequence of multiple adsorption/desorption cycles takes place inside the medium. Due to the internal mass transfer of molecules in the inter-fiber and at the surface of the medium fibers [17], the contact time in the medium is likely to be longer than the residence time calculated from geometrical considerations only. One assumption is that the air flow rate may interfere with the sequence of adsorption/desorption cycles and influence the overall performance of the PCO device. In addition, even though all experiments carried out in this study were based on the same PCO device and the same pollutant, it is probable that air flowed differently across the medium according to the operating conditions. As indicated in Figure 2, the presence of the UV lamps upstream of the medium and the configuration of the pleated medium itself could significantly change the air flow distribution along the filter and inside the material. Turbulence effects could be generated at high flow rates. However, from Figure 7, it can be assumed that the order of magnitude of the air velocity would not necessarily be influential because the theory is verified for the couple of velocities (v = 0.6 m/s and v = 1.9 m/s; case **{A}**) and the couple of velocities (v = 0.2 m/s and v = 1.0 m/s; case **{C}**), whereas it is not verified for the latter couple of velocities in the other cases. A numerical study of the air flow around and inside the medium seems necessary to investigate this point. Moreover, future studies using different filter geometries need to be performed to evidence the real influence of the flow rate.

### 4.4. Effect of the Initial Concentration

**Figure 8:**
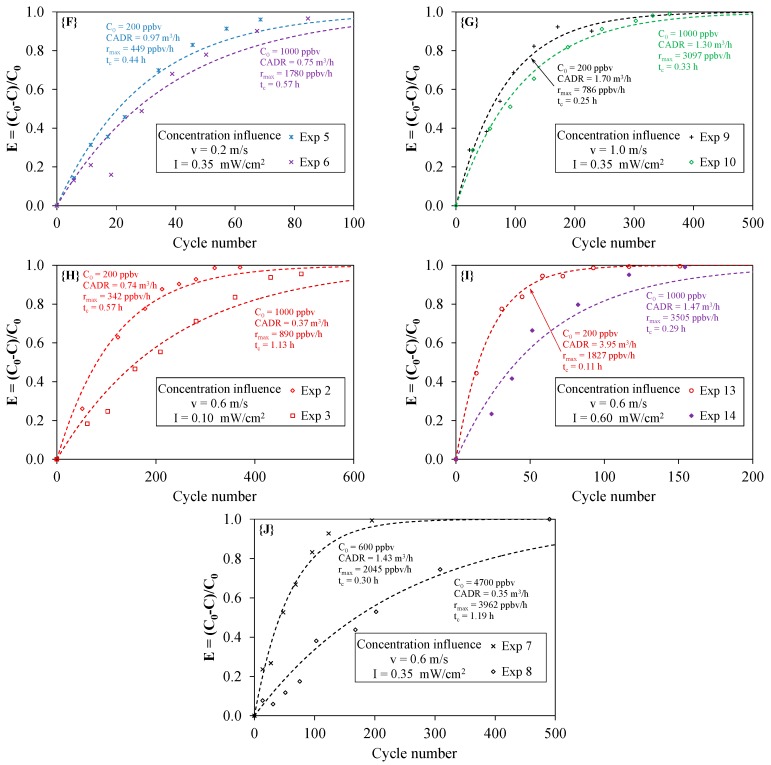
Effect of the initial concentration on toluene removal efficiency according to the light irradiance I and the air velocity v (cases **{F}**–**{J}**; experimental points and model; cycle number = 1/α).

For experiments carried out at a constant light irradiance and a constant air velocity, it is expected that the time needed for total toluene degradation should increase with the increase in the initial concentration. Figure 8 shows the effect of C_0_ on the determined parameters: CADR, time constant t_c_ and maximum degradation rate r_max_. Cases **{F} {G} {H}** and **{I}** enable a direct comparison between the results obtained since concentrations of 200 and 1000 ppbv were used in these four cases (corresponding to a concentration ratio of five). The results are contrasting. For I = 0.35 mW·cm^−2^ (cases **{F}** and **{G}**), the CADR values and the t_c_ values are of the same order of magnitude (ratio of 1.3), whereas for I = 0.10 mW·cm^−2^ (case **{H}**) and for I = 0.60 mW·cm^−2^ (case **{I}**), the ratios are equal to 2.0 and 2.7, respectively. Moreover, according to Figure 8, the maximum degradation rate r_max_ was higher for 1000 ppbv than for 200 ppbv, but the ratio r_max(1000 ppbv)_/r_max(200 ppbv)_ was around 4 for cases **{F}** and **{G}**, 2.6 for case **{H}** and only 2 for case **{I}**. In case **{J}** corresponding to a concentration ratio of eight due to the very high concentration used (4700 ppbv), the difference between CADR values was clear (ratio of four), but the maximum degradation rate obtained for this concentration (3962 ppbv/h) was not significantly higher than that obtained for a concentration of 1000 ppbv in other conditions. Actually, at this level of concentration, it is known that the reaction mechanism tends to a saturation reaction well described in the literature by the Langmuir–Hinshelwood kinetic rate law model [16]. It should be noted that cases **{H}** and **{J}** are based on Exp 3 and Exp 8, respectively, and thus, these results must be considered with caution (as discussed above). They confirm that it is difficult to determine experimentally the influence of a given parameter independently of the others [10]. Although these results highlight that the initial concentration is not the main parameter influencing the degradation of the pollutant, in comparison with the light irradiance, they nonetheless demonstrate that it is an important parameter that must be taken into account for the characterization of any PCO device. Consequently, with the aim of developing a reliable standard test method to assess the effectiveness of types of PCO apparatus, the given values of the initial concentration should be selected.

## 5. Conclusions

The performances of a laboratory PCO device were determined using a recirculation closed-loop pilot. The Clean Air Delivery Rate (CADR) of the apparatus was calculated under different operating conditions. The main results of this study are:

(i) Since the volume of the PCO device is very small in relation to the volume of the reservoir, the ratio of the residence times (τ_P_/τ_R_) tends to zero, and consequently, the concentration-time relationship given by Equation (1) can be used to model the experimental points. Moreover, as the term α is usually determined to be lower than 5%, the simplified model characterized by a first-order decay model (Equation (3)) can be used to determine the CADR of the PCO device.
(ii) Since the Excel^®^ solver is easily usable for data analysis, such a tool should be preferred for CADR determination rather than the analysis of the curve ln(C/C_0_) vs. time, which involves selecting a given number of points for a correct calculation.
(iii) According to the operating conditions, the CADR ranged from 0.35 to 3.95 m^3^·h^−1^, i.e., one order of magnitude for the same PCO device. The CADR was mainly dependent on the light irradiance intensity. An increase in the CADR with the square root of the light irradiance was observed.
(iv) Although the CADR of the PCO device inserted in the closed-loop reactor did not theoretically depend on the flow rate (see Part I), the experimental results did not enable the confirmation of this point. The results were contrasting. Some experimental data were in agreement with the theory, whereas others disagreed. Further investigations are therefore needed to explain this ambiguity. Numerical simulations of the air stream line and velocity through the medium may be useful.
(v) The maximum degradation rate r_max_ ranged from 342 to 4894 ppbv·h^−1^. As the initial concentration is one parameter influencing the degradation rate of the pollutant, tests should be performed at a given value of the initial concentration in order to compare the performances of different types of PCO apparatus.

Finally, this study demonstrated that a recirculation closed-loop pilot can be used to develop a reliable standard test method to assess the effectiveness of any PCO device with regard to the operating parameters. The conditions needed to apply a first-order decay model to the experimental data (described in Part I) were usually fulfilled. However, experimental errors in the determination of the pollutant concentrations still impact the assessment of the influential parameters too much. More accurate results should be obtained using the same operating conditions, and future research is still required to determine pollutant concentrations at low part-per-billion levels.

## Figures and Tables

**Table 1:** Operating conditions used in each experiment and experimental results.

	Experimental Conditions				Experimental Results						
	I (mW·cm^−^²)	v (m·s^−1^)	τ_R_ (s)	C_0_ (ppbv)	α (−)	R^2^	CADR (m^3^·h^−1^)	1/α (−)	t_c_ (h)	t_c_ (s)	r_max_ (ppbv·h^−1^)
Exp 1	0.10	0.2	52.5	600	0.0278	0.988	0.80	36	0.53	1917	1140
Exp 2	0.10	0.6	17.5	200	0.0086	0.973	0.74	117	0.57	2052	342
Exp 3	0.10	0.6	17.5	1000	0.0043	0.956	0.37	232	1.13	4065	890
Exp 4	0.10	1.0	10.5	600	0.0059	0.937	0.85	169	0.49	1781	1183
Exp 5	0.35	0.2	52.5	200	0.0337	0.969	0.97	30	0.44	1584	449
Exp 6	0.35	0.2	52.5	1000	0.0259	0.927	0.75	39	0.57	2055	1780
Exp 7	0.35	0.6	17.5	600	0.0166	0.971	1.43	60	0.30	1062	2045
Exp 8	0.35	0.6	17.5	4700	0.0041	0.964	0.35	244	1.19	4273	3962
Exp 9	0.35	1.0	10.5	200	0.0118	0.961	1.70	85	0.25	901	786
Exp 10	0.35	1.0	10.5	1000	0.0090	0.984	1.30	111	0.33	1171	3097
Exp 11	0.35	1.9	5.5	600	0.0047	0.966	1.29	212	0.33	1173	1841
Exp 12	0.60	0.2	52.5	600	0.0643	0.994	1.85	16	0.23	843	2639
Exp 13	0.60	0.6	17.5	200	0.0457	0.991	3.95	22	0.11	392	1827
Exp 14	0.60	0.6	17.5	1000	0.0170	0.929	1.47	59	0.29	1039	3505
Exp 15	0.60	1.0	10.5	600	0.0167	0.969	2.40	60	0.18	635	3971
Exp 16	1.00	0.6	17.5	600	0.0238	0.979	2.06	42	0.21	743	2934
Exp 17	2.00	0.6	17.5	600	0.0397	0.962	3.43	25	0.12	449	4894

## Abbreviations

A: cross-sectional area of the PCO device (m^2^)
C: pollutant concentration (mol·m^−3^)
CADR: Clean Air Delivery Rate (m^3^·s^−1^)
E: efficiency (dimensionless)
k: overall degradation rate constant (s^−1^)
L: length of the PCO device (m)
Q: flow rate (m^3^·s^−1^)
r: degradation rate (mol m^−3^·s^−1^)
t: time (s)
t_c_: time constant of the closed-loop reactor (s)
V_P_: PCO device volume (m^3^)
V_R_: reservoir volume (m^3^)
*Greek letters*
τ_P_: residence time in the PCO device (s)
τ_R_: residence time in the reservoir (s)
α: fractional yield of the treated flow rate (dimensionless)
